# The *Drosophila* blood–brain barrier invades the nervous system in a GPCR-dependent manner

**DOI:** 10.3389/fncel.2024.1397627

**Published:** 2024-05-23

**Authors:** Esteban G. Contreras, Steffen Kautzmann, Christian Klämbt

**Affiliations:** Multiscale Imaging Center, Institute of Neuro- and Behavioral Biology, University of Münster, Münster, Germany

**Keywords:** *Drosophila melanogaster*, blood–brain barrier, GPCR signaling, glial cells, cell processes

## Abstract

The blood–brain barrier (BBB) represents a crucial interface between the circulatory system and the brain. In *Drosophila melanogaster*, the BBB is composed of perineurial and subperineurial glial cells. The perineurial glial cells are small mitotically active cells forming the outermost layer of the nervous system and are engaged in nutrient uptake. The subperineurial glial cells form occluding septate junctions to prevent paracellular diffusion of macromolecules into the nervous system. To address whether the subperineurial glia just form a simple barrier or whether they establish specific contacts with both the perineurial glial cells and inner central nervous system (CNS) cells, we undertook a detailed morphological analysis. Using genetically encoded markers alongside with high-resolution laser scanning confocal microscopy and transmission electron microscopy, we identified thin cell processes extending into the perineurial layer and into the CNS cortex. Interestingly, long cell processes were observed reaching the glia ensheathing the neuropil of the central brain. GFP reconstitution experiments highlighted multiple regions of membrane contacts between subperineurial and ensheathing glia. Furthermore, we identify the G-protein-coupled receptor (GPCR) Moody as negative regulator of the growth of subperineurial cell processes. Loss of *moody* triggered a massive overgrowth of subperineurial cell processes into the CNS cortex and, moreover, affected the polarized localization of the xenobiotic transporter Mdr65. Finally, we found that GPCR signaling, but not septate junction formation, is responsible for controlling membrane overgrowth. Our findings support the notion that the *Drosophila* BBB is able to bridge the communication gap between circulation and synaptic regions of the brain by long cell processes.

## Introduction

One defining characteristic of living organisms involves the existence of barriers that delineate distinct compartments and tissues. As it is the case for many organs, the nervous system is protected by a specific barrier that effectively isolates neural tissue from the circulatory system. This so-called blood–brain barrier (BBB) is a particular intricate and highly selective barrier, regulating the influx and efflux of molecules, including ions and nutrients. Barriers generally have two important functions. On one hand they must block any paracellular diffusion and thus uncontrolled passage of solutes. On the other hand, the barrier must be equipped with specific transporters. Consequently, the precise control of all transport processes across the BBB is crucial for maintaining the proper function and health of the nervous system.

In higher vertebrates, the BBB is formed by endothelial cells of the vasculature within the nervous system. Paracellular diffusion is efficiently blocked by the establishment of tight junctions. The endothelial cells collaborate with pericytes and astrocytic endfeet to adjust transport of solutes across the BBB. Pericytes induce the formation of tight junctions and astrocytes function as vital connectors, bridging the gap between the BBB and the metabolic requirements of the neurons (Daneman et al., 2010; Bélanger et al., 2011; Liu et al., 2018). Collectively, they constitute what is known as the neurovascular unit (Daneman and Prat, 2015; Sweeney et al., 2019; Schaeffer and Iadecola, 2021).

**Figure 1:**
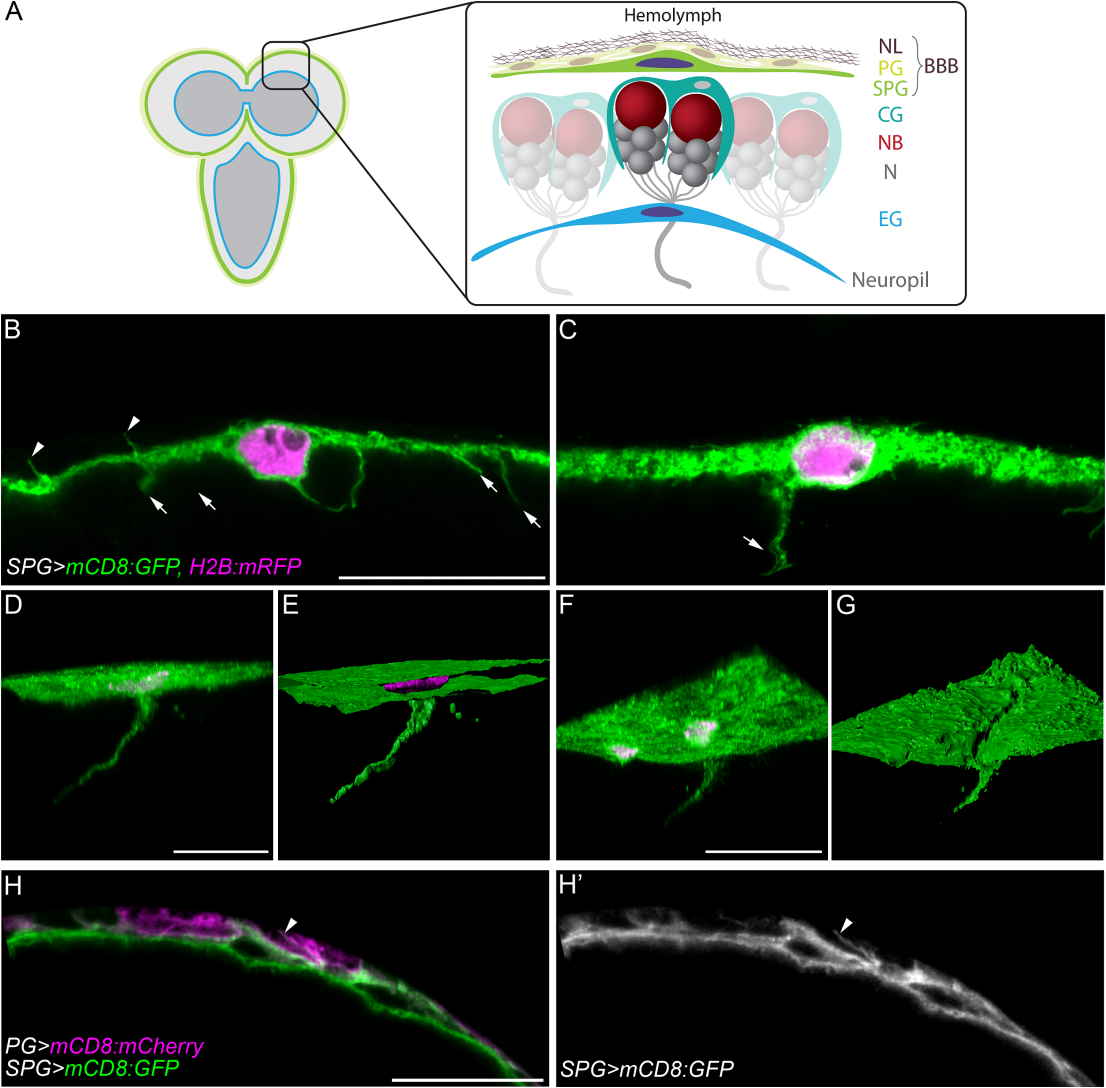
Cell processes extend from subperineurial glial cells of the blood-brain barrier. **(A)** A schematic representation of the larval brain and the main cell types present in the cortex and the blood-brain barrier. BBB, blood-brain barrier; CG, cortex glia; EG, ensheathing glia; N, neuron; NB, neuroblast; NL, neural lamella; PG, perineurial glia; SPG, subperineurial glia. **(B,C)** High-resolution confocal microscopy images of *mdr65-GAL4, UAS-mCD8:GFP, UAS-H2B:mRFP* third instar larval brains. **(C)** Corresponds to a z-projections of 5 optical sections. **(D,F)** Three-dimensional views of z-stacks from *mdr65-GAL4, UAS-mCD8:GFP, UAS-H2B:mRFP* third instar larval brains. **(E,G)** Surface reconstruction of the image channels in **(D,F)**. Subperineurial glial cell membranes are marked by mCD8:GFP (green) and nuclei by H2B:mRFP (magenta). **(H–H´´)** Single high-resolution optical section image of *apt^E01^-GAL4, mdr65-LexA, UAS-mCD8:mCherry*, lexAop-mCD8:GFP. Perineurial glial cell membranes are marked in magenta and Subperineurial glial cells in green. Arrows indicate cell processes going to the brain cortex, while arrowheads show cell processes to the perineurial glia. Scale bars are 20 µm.

A blood–brain barrier is found throughout the evolution of complex nervous systems (Bullock and Horridge, 1965). Within invertebrate phyla such as arthropods it has been particularly well studied and closely mirrors the functionality of the vertebrate BBB, both in maintaining homeostasis and responding to disease (Lane, 1991; Carlson et al., 2000; Dunton et al., 2021; Contreras and Sierralta, 2022; Contreras and Klämbt, 2023). Moreover, primitive vertebrates as well as invertebrates utilize glial cells to establish the BBB (Lane, 1991; Bundgaard and Abbott, 1992, 2008; Lane and Abbott, 1992). *Drosophila melanogaster*, the fruit fly, stands as the most extensively studied invertebrate BBB model. Insects possess an open circulatory system with a heart valve that pumps hemolymph, their equivalent blood, to all organs, including the nervous system (Tao and Schulz, 2007). Therefore, the *Drosophila* BBB has evolved to surround the neural tissue, effectively separating it from the circulatory system. The fly BBB consists of two layers of glial cells and, sitting on top, a dense extracellular matrix known as the neural lamella (Stork et al., 2008; Hindle and Bainton, 2014; Limmer et al., 2014). The paracellular barrier is formed by a layer of subperineurial glia (SPG) that establish pleated septate junctions as occluding cell–cell junctions (Tepass and Hartenstein, 1994; Carlson et al., 2000; Schwabe et al., 2005; Stork et al., 2008). They are the functional equivalent of vertebrate tight junctions and in part harbor related proteins (Behr et al., 2003; Nitta et al., 2003; Wu et al., 2004; Stork et al., 2008; Jaspers et al., 2012; Petri et al., 2019). The perineurial glial cells (PG), a group of star-like shaped glial cells, occupy the interface between the subperineurial barrier and the neural lamella, which is in direct contact with the hemolymph (Stork et al., 2008; Yildirim et al., 2019; see Figure 1A). Through the use of confocal and transmission electron microscopy, subperineurial glia have been characterized as a very flat, simple squamous epithelium (Schwabe et al., 2005, 2017; Silies et al., 2007; Awasaki et al., 2008; Stork et al., 2008). The subperineurial glia contact the perineurial glia on the humoral side (facing the hemolymph). On the neural side, they first interact with neuroblasts, which are the *Drosophila* neural stem cells. As larval development progresses, subperineurial glial cells make contact with the cortex glial cells that surround and separate neuroblasts and their neuronal progeny (Coutinho-Budd et al., 2017; Spéder and Brand, 2018; Rujano et al., 2022; Banach-Latapy et al., 2023).

Subperineurial glial cells are formed during late embryogenesis, when they first cover the entire nervous system (Carlson and Hilgers, 1998; Schwabe et al., 2005, 2017). Later in larval development, subperineurial glial cells undergo endomitosis and become multinucleated and polyploid in order to maintain the integrity of the BBB during fast CNS growth (Unhavaithaya and Orr-Weaver, 2012; Von Stetina et al., 2018; Zülbahar et al., 2018). Throughout development, these cells adjust their cell size to the growing CNS. They never divide and stay intact from embryonic to adult stages (Awasaki et al., 2008; Stork et al., 2008; Winkler et al., 2021).

Soon after subperineurial glial cells have been formed during late embryonic stages they start to establish septate junctions to block paracellular diffusion. An important regulator of septate junction formation is the orphan G protein-coupled receptor (GPCR) Moody (Bainton et al., 2005; Schwabe et al., 2005). In subperineurial glia, Moody signaling regulates the actomyosin cytoskeleton and via PKA affects subperineurial glia polarity (Hatan et al., 2011; Li et al., 2021). Loss of *moody* results in fragmented and frayed septate junction strands (Babatz et al., 2018). Interestingly, although fragmented septate junction strands do not efficiently block paracellular diffusion, *moody* mutants survive to adulthood. This can be explained by compensatory cell–cell interdigitations that increase the length of the paracellular diffusion path which, thus, re-establish an efficient barrier (Bainton et al., 2005; Babatz et al., 2018). These findings demonstrate that subperineurial glial cells are able to adjust their morphology to specific barrier needs.

To better understand the plastic behavior of the subperineurial glial cells, we conducted a comprehensive analysis of their morphology in larval *stages* using genetically encoded markers alongside with high-resolution laser scanning confocal microscopy and transmission electron microscopy (TEM). By marking subperineurial glia membranes, we showed that the typical flattened shape of subperineurial glial cells is interrupted by the emergence of short cell processes to both sides, humoral and neural, of the BBB. Remarkably, a small group of these processes are able to extend across long distances, navigating between the brain cortex and reaching into close proximity to the neuropil glia. Interestingly, *moody* mutants show a massive overgrowth of these membrane protrusions, that now form sheet-like structures that invade the brain cortex. Further mutant analyses demonstrate that the formation of these cell protrusions is under the control of the GPCR signaling pathway and reflects disruption in cell polarity.

## Results

### Subperineurial glial cells extend long processes into the central brain

The *Drosophila* BBB is a flat and thin barrier composed of two distinct layers of glial cells (Awasaki et al., 2008; Stork et al., 2008; Schwabe et al., 2017). To investigate the morphology of the BBB, we marked subperineurial glial cells (SPG) using the *mdr65-GAL4* driver in combination with two fluorescent markers, mCD8:GFP and H2B:mRFP, labelling subperineurial glial cell membranes and nuclei, respectively. Using high-resolution confocal microscopy, the thickness of the remarkably flat larval subperineurial glial cells was determined between 1.5 to 2 μm. This analysis also identified numerous thin cell processes of subperineurial glial cells with a length between 2.5–8 μm. They project either into the neural cortex of the larval brain (arrows in Figures 1B,C), or towards the humoral side of the brain intermingling between the perineurial glial cells (arrowheads in Figures 1B,H,H,’).

Interestingly, by generating three-dimensional reconstructions of a high resolution confocal z-stacks, we identified a small subset of cell processes that extended over long distances into the central brain cortex (Figures 1D,E), reaching an extension length of up to 75 μm. These processes initiated as flat sheet-like structures and tapered into extremely thin cell protrusions measuring around 0.3–0.4 μm of diameter (Figures 1F,G). While small cell processes were distributed throughout the subperineurial glia surface in the larval brain and ventral nerve cord, longer cell processes were relatively scarce (typically 1–2 per brain lobe) and were primarily located in the anterior region of the larval central brain.

**Figure 2:**
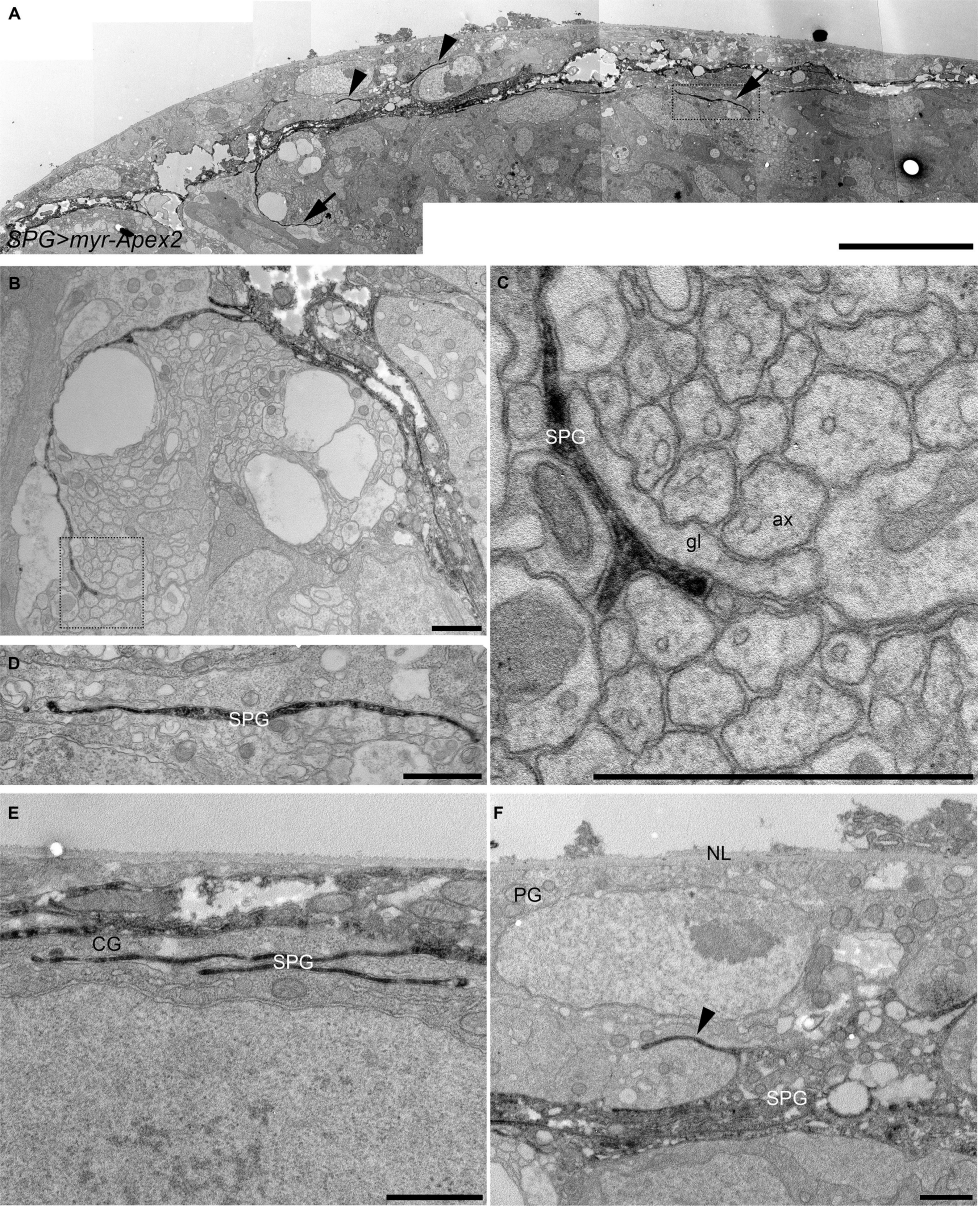
Electron microscopy analysis of subperineurial glia processes. Peroxidase-based electron microscope sections of third instar larval brains labelling subperineurial glial cells using *moody-GAL4, UAS-myr-Apex2*. **(A)** Aligned tiles of the larval blood–brain barrier. Arrows indicate cell processes going to the brain cortex, while arrowheads show cell processes directed towards the perineurial glia. **(B–F)** Subperineurial glial cell processes that contact axons and glial cells. **(C,D)** are enlarged images of the square region in **(A,B)** respectively. **(E)** Multiple cell processes below the BBB contacting only a cortex glial cell. **(F)** Shows a cell process projecting into the perineurial glial cell layer. Three larval brains were analyzed. ax, axon; CG, cortex glia; g, glia; NL, neural lamella; SPG, subperineurial glia; PG, perineurial glia. Scale bars are 10 μm **(A)** and 1 μm **(B–F)**.

To confirm that the presence of these membrane projections was not an artefact of confocal microscopy and to obtain a higher resolution, we adopted a peroxidase-based electron microscopy approach. We expressed a membrane-tagged form of the Apex2 peroxidase (Rey et al., 2023) in all subperineurial glia using the *moody-GAL4* driver (Bainton et al., 2005; Stork et al., 2008). Single electron microscopy sections revealed numerous cell processes originating from the subperineurial layer and extending into the cortex (Figure 2). Remarkably, some of these processes are in contact with axons and glial cell processes (Figures 2B–D), whereas others appear to have only glial cell contact (Figure 2E). Additionally, we also observed small cell processes extending from the humoral side of subperineurial glia in between perineurial glia (arrowheads in Figures 2A,F). Altogether this evidence supports the notion that subperineurial glial cells, while mostly flat in appearance, are able to extend fine processes that reach out to neighboring cells and structures within the larval central brain.

### Subperineurial glia processes navigate through the brain cortex

**Figure 3:**
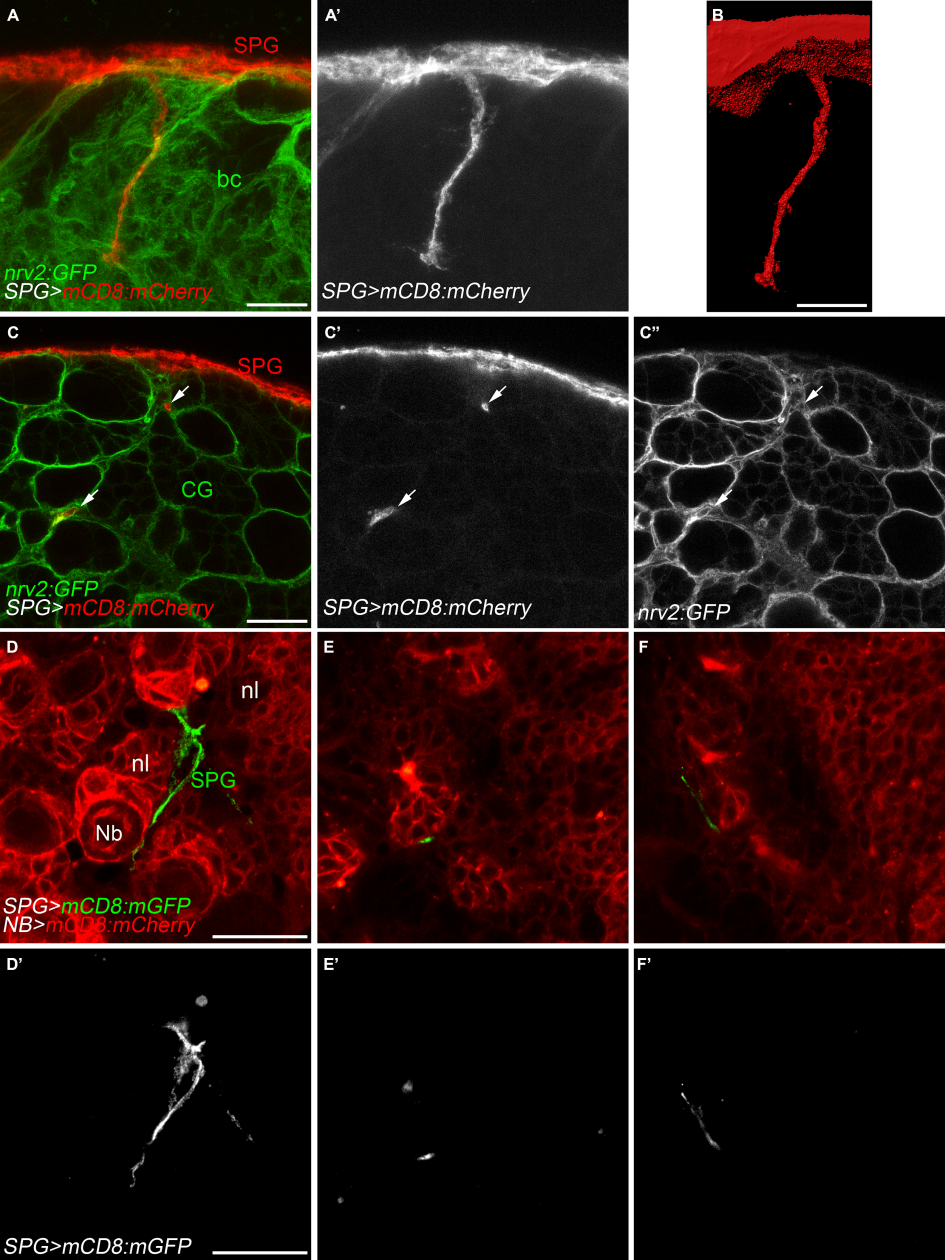
Subperineurial glial cell processes navigate in between cortex glial cells. **(A–C’’)** High-resolution confocal images of third instar larval brains of *nrv2:GFP*, *moody-GAL4*, *UAS-mCD8-mCherry* animals. Nrv2:GFP marks all cortex glial cell membranes (green) and mCD8:mCherry labels subperineurial glial cell membranes (red). **(A,A’)** z-projections of 5 optical sections. **(B)** Surface reconstruction of the membrane marker in (**A,A’**, mCD8:mCherry). **(C–C’’)** Single high-resolution optical sections of the brain cortex. Arrows show cell processes projecting in-between cortex glial cell chambers. **(D–F’)** Larval brains of *wor-GAL4*, *moody-LexA*, *UAS-mCD8:mCherry*, *lexAop-mCD8:GFP* animals. Subperineurial glial cell membranes are labelled in green or gray, while neuroblast and neuronal membranes are marked in red. Images are single high-resolution optical sections of the same *z*-stack from the surface of the larval brain going into the neuropil. bc, brain cortex; CG, cortex glia; Nb, neuroblast; nl, neuronal lineage; SPG, subperineurial glia; PG, perineurial glia. Scale bars are 20 μm.

During first instar larval development, subperineurial glial cells directly contact actively dividing neuroblasts (Coutinho-Budd et al., 2017; Spéder and Brand, 2018; Rujano et al., 2022). At late larval development, subperineurial glia promote reactivation of neuroblast division through the secretion of insulin-like peptides, and consequently, initiating secondary neurogenesis (Chell and Brand, 2010; Sousa-Nunes et al., 2011; Spéder and Brand, 2018). Given that subperineurial glia send large cell processes into the cortex, we questioned whether this processes could infiltrate the cortex glial ‘*trophospongium’*, a membrane structure that encases neuroblasts lineages and their neuronal progeny forming individual chambers (Hoyle et al., 1986; Spéder and Brand, 2018), in order to maintain contact with the neurogenic niche. We used a *nrv2:GFP* protein trap insertion to label cortex glial membranes and mCD8:RFP to mark subperineurial glia membranes. Interestingly, we observed that subperineurial glial cells project long cell processes deeply through the cortex glia (Figures 3A,B). However, these processes extend across narrow canaliculi in between the cortex glia membranes, without entering the individual glial chambers that encase the neuroblast and its progeny (Figures 3C–C’’).

In order to directly label neuroblasts and their lineages, we employed the *wor-GAL4* driver (Albertson et al., 2004), while subperineurial glia membranes were marked using the LexA system (*moody-LexA*, *lexAop-mCD8:GFP*). Similar to our previous approach, subperineurial glia cell processes navigated between the neuroblast lineages without infiltrating individual neuronal cell lineages (Figures 3D–F’). These results suggest there is no direct contact between subperineurial glia cell processes and the neuronal cell bodies.

### Subperineurial and ensheathing glial cells form direct contacts

**Figure 4:**
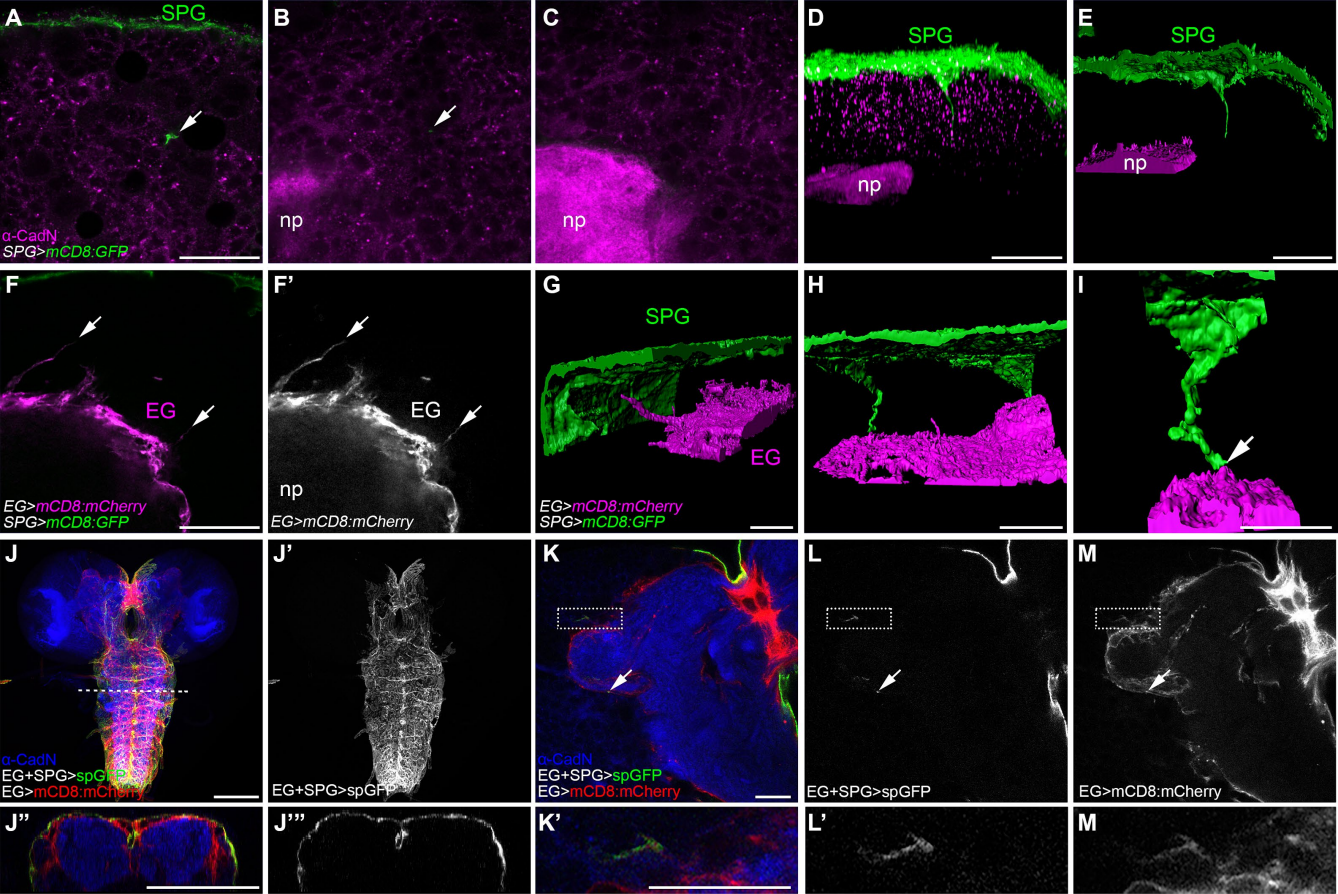
Subperineurial long cell processes encounter with ensheathing glia without infiltrating de neuropil. **(A–E)** Third instar larval brain high-resolution images of *moody-GAL4, UAS-mCD8:GFP* animals stained for the neuropil marker Cadherin-N (CadN, magenta). **(A–C)** Single confocal microscopy planes of the same z-stack at different positions. Arrows show a subperineurial glial cell process close to the neuropil. **(D,E)** Three-dimensional reconstructions of z-stack in **(A–C)**. **(D)** corresponds to fluorescence signal view, while **(E)** to surface reconstruction. **(F–I)** Third instar larval brain high-resolution images of *moody-LexA*, *R83E12-GAL4*, *lexAop-mCD8:GFP*, *UAS-mCD8-mCherry* animals. **(F,F’)** Single confocal plane showing ensheathing glial cell processes (arrows). **(G–I)** Three-dimensional surface reconstructions showing subperineurial glial cell processes (green) and ensheathing glial cell processes (magenta). Arrow points to a subperineurial glial cell process in close proximity to ensheathing glia. **(I)** Corresponds to a magnified reconstruction of the brain in **(H)**. **(J–M’)** splitGFP (spGFP) reconstitution in larval brains of *R83E12-GAL4*, *mdr65-LexA*, *UAS-CD4:spGFP1-10*, *lexAop-CD4:spGFP11*, *UAS-mCD8:mCherry* stained against CadN (blue). **(J”,J”’)** are orthogonal sections of the VNS at the dashed line in **(J)**. **(K’,L’,M’)** are enlarged images of the square region in **(K)**. Interaction between subperineurial- and ensheathing glial cells are seen in green, all ensheathing glial cell membranes in red. Arrows show spGFP reconstitution in the central brain neuropil. **(J–J”’)** Correspond to normal confocal microscopy and **(K–M’)** are high-resolution confocal microscopy images. EG, ensheathing glia; np, neuropil; SPG, subperineurial glia. Scale bars are 100 μm in **(J,J”’)** and 20 μm in **(A–I,K–M’)**.

The long cell processes emerging from the subperineurial glia do not establish direct contact with neuronal cell bodies within the brain cortex, instead, they navigate in between lineages encapsulated by the cortex glia (Figure 3). To test the hypothesis that the final target of the subperineurial glial processes is the brain neuropil, we stained for the localization of *Drosophila* N-Cadherin (CadN), a neuronal adhesion protein known to accumulate at central nervous system neuropils (Iwai et al., 1997). Interestingly, long cell processes of the subperineurial glia never infiltrated the neuropil, but stopped in close proximity (Figures 4A–E). This led us to consider whether these processes may interact with ensheathing glial cells. These glial cells surround the entire neuropil and isolate it from the cortex by establishing an internal diffusion barrier (Pereanu et al., 2005; Otto et al., 2018; Pogodalla et al., 2021). We therefore labelled the cell membranes of the ensheathing glia using the *R83E12-GAL4* driver, while marking the subperineurial glia using the LexA system (*moody-LexA*, *lexAop-mCD8:GFP*). Surprisingly, we found that the ensheathing glia also extend cell processes towards the subperineurial glial cells (Figures 4F,G). Likewise, long processes of the subperineurial glia appeared to reach the ensheathing glial layer (Figures 4H,I).

To more specifically assess whether processes of the two glial cell populations, the subperineurial glial cells and the ensheathing glial cells, have direct contact, we used a modification of the *GFP Reconstitution Across Synaptic Partners (GRASP)* method initially developed to detect synaptic contacts (Feinberg et al., 2008; Bosch et al., 2015). Here, one domain of GFP (spGFP^1-10^) is expressed under UAS control at the subperineurial glial cell membrane while the complementary domain (spGFP^11^) is expressed at the plasma membrane of the ensheathing glial cell under LexAop control. GFP fluorescence is only reconstituted where both GFP protein fragments meet and complement. In addition, we stained the neuropil for CadN localization, and labelled the ensheathing glial cell membranes by the expression of mCD8:mCherry. We observed strong spGFP signal in the dorsal and lateral regions of the larval ventral nerve cord and the large commissure connecting the two brain lobes (Figures 4J–J”’). This demonstrates that subperineurial glia are able to interact with ensheathing glia. When tested for such interaction in the central brain, GFP reconstitution was neither observed across the surface of the ensheathing glia nor inside the neuropil. However, weak, spotted signals could be found sparsely along the ensheathing glial cell membrane facing the central brain cortex (Figures 4K–M’). All these results suggest the presence of extensive physical interactions between subperineurial glial cells of the blood–brain barrier and the ensheathing glial cells encasing the neuropil of the *Drosophila* larval CNS.

### Loss of GPCR signaling induces membrane overgrowth

A key regulator of subperineurial glia growth and septate junction formation during embryonic development, is the orphan G protein-coupled receptor (GPCR) Moody (Bainton et al., 2005; Schwabe et al., 2005, 2017). To investigate how the formation of cell processes of subperineurial glia is regulated during development, we chose to analyze membrane growth in animals lacking the *moody* gene (*moody^ΔC17^*) (Bainton et al., 2005; Schwabe et al., 2005). Upon crossing homozygous mutant *moody* females with males carrying a subperineurial glia GAL4 driver insertion to label all membranes (*mdr65-GAL4, UAS-mCD8:GFP*), all female offspring carry one functional copy of *moody*, while all male offspring are hemizygous *moody* mutant.

**Figure 5:**
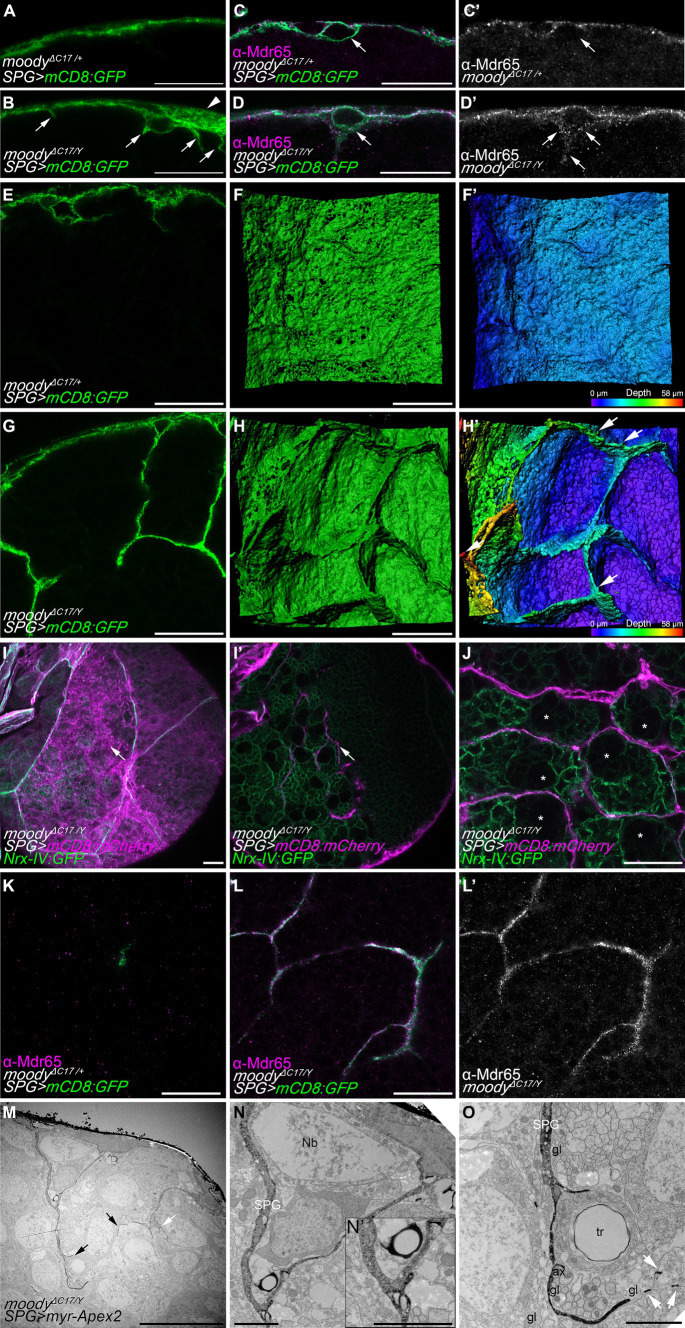
Loss of *moody* induces membrane overgrowth. **(A,B)** Single high-resolution optical sections of *moody^ΔC17/+^* heterozygous **(A)** and *moody^ΔC17/Y^* hemizygous **(B)** third instar larval brains. Subperineurial glial cells are labelled by crossing mutants to *mdr65-GAL4, UAS-mCD8:GFP* (green). Arrowhead points to membrane overgrowth where luminal and neural domains of the subperineurial glial cells are separated more clearly. Arrows show cell processes projecting into the cortex. **(C–D’)** High-resolution confocal images of larval brain of *moody^ΔC17/+^* **(C,C’)** and *moody^ΔC17/Y^* **(D,D’)** animals crossed to *mdr65-GAL4, UAS-mCD8:GFP* (green) and stained for the localization of the Mdr65 protein (magenta and grey). Arrows show the neural side of the subperineurial glial cell. **(E,G)** Cross section of a larval brain of *moody^ΔC17/+^* and *moody^ΔC17/Y^* mutant showing long cell processes infiltrating the cortex. **(F,F’,H,H’)** Three-dimensional surface reconstructions of the BBB in **(E,H)** labelled in green **(F,H)** and in pseudo color gradient representing the position in the *z* axis **(F’,H’)**, showing the formation of chamber-like structures. **(I–J)** Confocal sections of third instar larval brains of *moody^ΔC17/Y^* mutants. Subperineurial glial cells are marked by *mdr65-GAL4, UAS-mCD8:mCherry* (magenta) and septate junctions and cortex glial cell membranes using *nrx-IV:GFP* allele (green). **(I)** Is a normal confocal image of superficial view of the subperineurial layer (magenta) showing septate junctions (green) to demark cell boundaries. **(I’)** Is a deeper section in the cortex of the same larval brain in **(I)**. Arrows show processes infiltrating the cortex that does not originate from the boundaries between two subperineurial glial cells. **(J)** Single high-resolution confocal section of the brain cortex showing subperineurial glial cell membrane sheets surrounding the trophospongium formed by the cortex glial cells. Neuroblasts positions are highlighted by stars. **(K–L’)** High-resolution confocal images of *moody^ΔC17/+^* heterozygous **(K)** and *moody^ΔC17/Y^* hemizygous **(L,L’)** third instar larval brain cortex stained for Mdr65 protein (magenta) and SPG membrane marked using *mdr65-GAL4, UAS-mCD8:GFP*. Numbers of larval brains “n” are 4 **(A,E,F,F’)**, 7 **(B,G,H,H’)**, 10 **(C–D’,K–L’)** and 5 **(I,J)**. **(M–O)** Sections of *moody^ΔC17^* hemizygous larval brains using peroxidase-based electron microscopy. Subperineurial glial cell are marked using *moody-GAL4, UAS-myr-Apex2*. Three larval brains were analyzed. **(N,O)** High magnification images of labelled membrane projections in the brain shown in **(M)**. Arrows indicate cell processes going to the brain cortex, white arrows show small membrane fragments suggesting the presence of thin filopodia-like structures. ax, axon; gl, glia; Nb, neuroblast; SPG, subperineurial glia; PG, perineurial glia; tr, trachea. Scale bars are 20 μm in **(A–L’)**, 10 μm in **(M)**, and 2 μm in **(N,O)**.

Interestingly, the subperineurial glia of hemizygous *moody^ΔC17/Y^* mutants presented large membrane overgrowth areas that were not observed in heterozygous animals (*moody^ΔC17/+^*) (Figures 5A,B). Moreover, several cell processes were found to extend from the subperineurial glial cells towards the CNS cortex (Figures 5A,B). These findings suggest that loss of *moody* function promotes an excess of cell growth and indicate a function in cell polarity. As Moody has been shown to localize at the neural side of the subperineurial glia (Mayer et al., 2009; Li et al., 2021), we further tested whether loss of *moody* affected subperineurial glial cell polarity in larval brains. We focused on the localization of the xenobiotic transporter Mdr65, which is enriched at the humoral side of wild type subperineurial glial cells (Figures 5C,C’; Mayer et al., 2009). Interestingly, in *moody* mutant brains, the Mdr65 protein is evenly localized across both plasma membrane sides, humoral and neural, suggesting a defect in the polarization of localized components of the BBB plasma membrane (Figures 5D,D’).

In addition, loss of *moody* also triggered an overgrowth of cell processes into the brain (Figures 5B,G). Upon z-stack reconstruction, we observed that large extensions of flat membrane sheets project from the subperineurial glia into the cortex, forming chamber-like structures (Figures 5E–H’). These structures resemble individual cortex glia chamber structures (Dumstrei et al., 2003; Pereanu et al., 2005; Spéder and Brand, 2018) and were not observed in control animals. Next, we determined whether these membrane sheets originated at the cell–cell junctions between two individual subperineurial glial cells. For this, we labeled the septate junctions formed at the junctions of subperineurial glial cells using the *nrx-IV:GFP* protein trap (Edenfeld et al., 2006) in conjunction with a general subperineurial membrane marker (mCD8:mCherry in red). In *moody* mutant larval brains, cell processes did not extend from the boundaries between two cells (see arrows in Figures 5I,I’), indicating they did not project from the septate junction regions. Instead, these membrane sheets surrounded the chamber pattern generated by cortex glial cells (Figure 5J), suggesting they behave similarly to the long cell protrusions present in wild-type larval brain.

In the membrane sheet structures that infiltrate the cortex of *moody* mutants, we also found intensive Mdr65 localization which was never detected within the brain of heterozygous control animals (Figures 5K–L’). This furthermore demonstrates that in *moody* mutants subperineurial glial cell polarity is indeed disrupted. To further validate the presence of excessive subperineurial cell processes in the brain of *moody* mutants, we proceeded to analyze *moody^ΔC17^* mutant animals using a peroxidase-based staining method for electron microscopy as it was previously performed in control animals. In accordance with what was observed in high-resolution confocal microscopy, *moody* mutant animals displayed excessive membrane protrusions within the cortex (arrows in Figure 5M). These processes surround neuronal cell bodies and reach axons fascicles (Figures 5N,O). Two cell processes originating from the subperineurial glia made contact in the cortex region (Figure 5N’), suggesting that they may be generating chamber-like structures as it was observed using high-resolution confocal microscopy.

**Figure 6:**
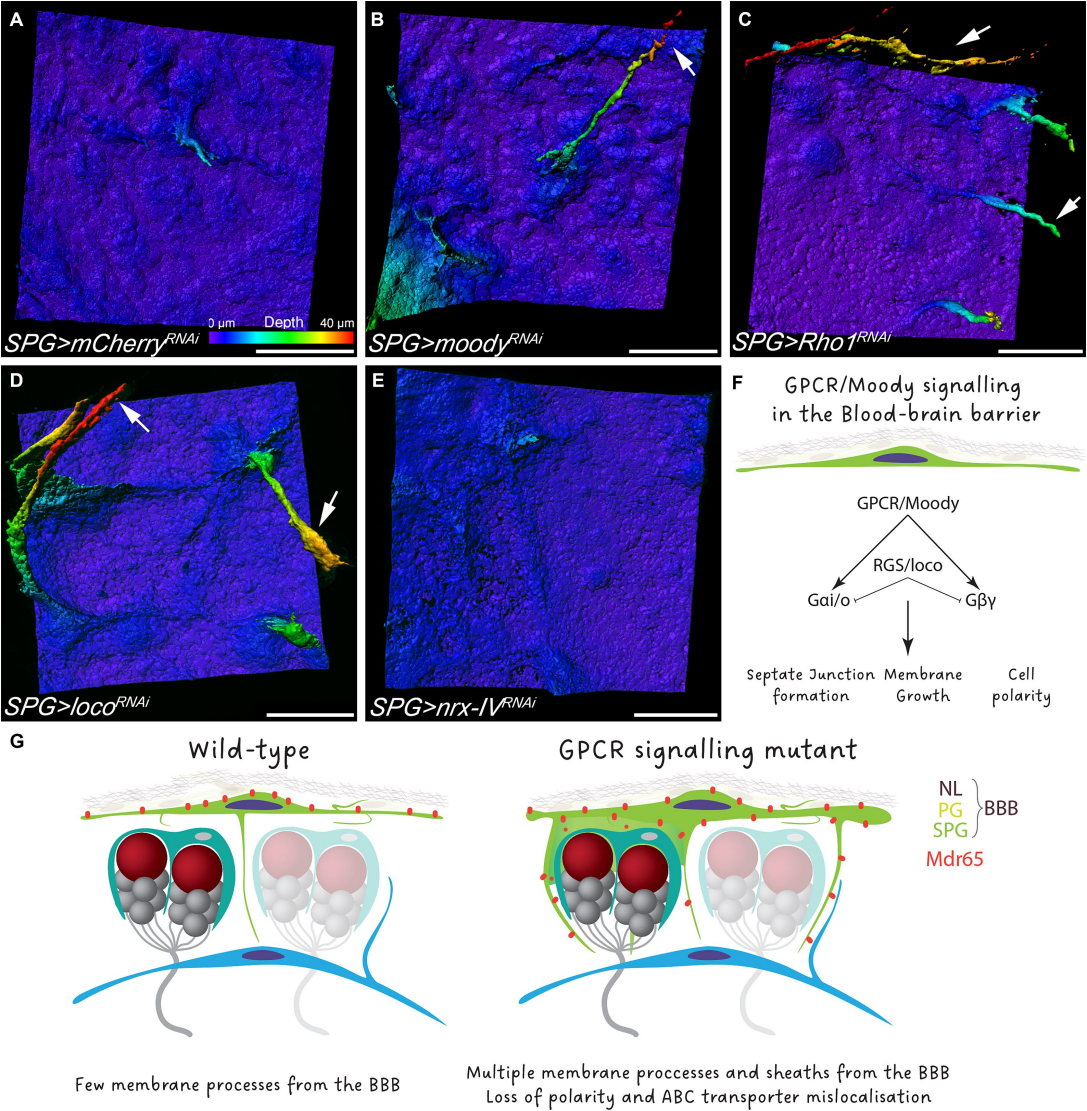
GPCR signaling regulate polar membrane extension in subperineurial glial cells. **(A–E)** Three-dimensional surface reconstructions of third instar larval subperineurial glial cells upon RNAi-mediated knockdown of **(A)** *mCherry* as control, **(B)** *moody*, **(C)** *Rho1*, **(D)** *loco* and **(E)** *nrx-IV* driven by *mdr65-GAL4, UAS-mCD8:GFP*. Arrows show long cell processes originating form subperineurial glial cells. Pseudocolor gradient represents the position in the *z* axis. Numbers of larval brains “n” are 20 **(A)**, 4 **(B)**, 9 **(C)**, 11 **(D)** and 10 **(E)**. Scale bars are 20 μm. **(F)** Schematic representation of the GPCR signaling in subperineurial glial cells and the affected processes. **(G)** Working model of the *Drosophila* BBB.

Moody has been characterized as a GPCR that controls actomyosin contractility and the proper formation of septate junctions (Bainton et al., 2005; Schwabe et al., 2005; Hatan et al., 2011; Babatz et al., 2018; Li et al., 2021). To understand whether GPCR signaling is necessary in subperineurial glia to block the formation of membrane sheets, we knocked down *moody* in subperineurial glial cells in a cell-autonomous manner. Similar to *moody* mutants, RNAi-mediated knockdown of *moody* induced the formation of multiple long processes, however, extended membrane sheets were much less frequent as noted in *moody* mutants (Figures 6A,B). Equivalent phenotypes were observed when the GPCR signaling regulator Loco (Granderath et al., 1999), or the downstream target and key regulator of the actin cytoskeleton Rho1 (Li et al., 2021), were knocked down in subperineurial glial cells (Figures 6C,D).

Given that GPCR signaling regulates the formation and maintenance of septate junction strands (Schwabe et al., 2005; Babatz et al., 2018), we also wanted to assess if the subperineurial glia membrane overgrowth observed upon loss of *moody* function is a consequence of the disruption in septate junction formation and thus, a disruption of blood–brain barrier integrity. Therefore, we silenced expression of *nrx-IV*, that encodes a core component of septate junctions, expression using RNA interference (Baumgartner et al., 1996). In such animals, blood–brain barrier integrity is compromised, but animals can survive until late larval stages due to compensatory subperineurial glial cell–cell interdigitations (Babatz et al., 2018). Upon subperineurial glial cell specific knockdown of *nrx-IV*, no extensive formation of membrane protrusions into the CNS was observed (Figure 6E). This suggests that GPCR signaling regulates septate junction formation and membrane growth independently from each other (Figure 6F). Altogether, these results indicate that loss of GPCR signaling in the BBB promotes membrane overgrowth that culminates in the invasion of the brain cortex by the BBB.

## Discussion

In this study, we have shown that subperineurial glial cells extend cell processes of varying length into both the humoral and neural side of the larval brain. While long cell processes navigate in between cortex glial cells towards the ensheathing glia encasing the neuropil, they are unable to invade the neuropil itself. Both, at the dorsal ventral nerve cord as well as the medial posterior side of the central brain, the subperineurial glial cells are in direct contact with the ensheathing glia that cover all neuropils. Importantly, the formation of these processes does not appear to be linked to the tightness of the blood–brain barrier (BBB) but rather is regulated by GPCR signaling. Thus, our data support a model in which the *Drosophila* BBB not only envelops the entire nervous system but also establishes direct connections with the neuropil glia.

Through the use both high-resolution microscopy and electron microscopy in combination with a peroxidase-based staining method, we observe cell processes originating from subperineurial glial cells in the immediate vicinity of the brain neuropil and axon bundles. This aligns with previous observations by transmission electron microscopy of the larval peripheral nerves (Stork et al., 2008), supporting the notion that the subperineurial layer does not constitute simple flat endothelium-like structure, but rather develops numerous cell processes that establish contacts to all other cell types in the brain. Short processes from subperineurial glial cells have been noted in the central brain (Li et al., 2021). However, the lack of a subperineurial glia marker when analyzing electron microscopy sections made it challenging to recognize long cell processes infiltrating the brain cortex, as they only represent very thin and narrow membrane protrusion within a cortex teeming with glial membranes.

The molecular processes that regulate the formation of these cell processes remain unclear. Moreover, it is plausible that they behave as dynamic structures that extend and retract throughout development, depending on physiological needs. We demonstrated that the extension of cell processes is under the control of the G protein-coupled receptor Moody, as the loss of *moody* resulted in the presence of massive membrane sheets projecting into the cortex. This phenotype corresponds to an exacerbated growth given than in control situations only few projections can be found (Figure 6G).

The establishment of the polarity of subperineurial glial cell is not fully understood, however, it is proposed that contact with the basal lamina during embryogenesis is the initial polar cue (Schwabe et al., 2017). Most apico-basal polarity markers such as phosphoinositides, Par and Crumbs complexes, are absent in subperineurial glial cells. *Moody* mutant subperineurial glial cells lose polarity and the neural domain is not properly established and thus likely non-functional. This may be associated with a requirement to massively extend membrane protrusions into the neural cortex. Loss of Protein kinase A (PKA) function has been shown to mislocalize Moody protein, also affecting subperineurial glial cell polarity (Li et al., 2021). Furthermore, *moody* mutant animals exhibit increased membrane cell–cell interdigitations as a compensatory mechanism for the fragmentation of septate junctions (Babatz et al., 2018). An analogous compensatory mechanism may account for the excess of cell processes generated in a *moody* mutant background. The absence of proper subperineurial glial cell polarity, which is mirrored by an altered Mdr65 localization, could also impact the localization of nutrient or ion transporters, as well as, correct glial-glial or glial-neuronal communication. A BBB dysfunction in the transport of macromolecules could potentially be compensated by an increase in subperineurial glia membrane growth and the contact with other glial cell types. This conception is supported by the fact that *nrx-IV* knockdown, that affects septate junction formation and paracellular transport, does not affect membrane growth, suggesting that GPCR signaling regulates both paracellular and transcellular permeability through independent mechanisms.

The role of cell processes originating at the neural face of the subperineurial glia remains speculative. We endorse the notion that they serve as bridges between the blood–brain barrier and inner neural tissues. Likewise, a potential role of small process is the increase in contact surface between subperineurial and cortex glia. Communication between glial subtypes has been proposed to be mediated by gap junctions (Holcroft et al., 2013; Volkenhoff et al., 2018; Zhang et al., 2018; Weiss et al., 2022). Furthermore, in the peripheral nerves, gap junctions also mediate cell–cell adhesion between subperineurial glial cells and wrapping glial cells (Kottmeier et al., 2020; Das et al., 2023). Therefore, cell processes may function as metabolic bridges between different glial cell types. Regarding the function of long cell processes, we propose that they could interact with ensheathing glial cell. This is supported by the observation that the ensheathing glia can also extend cell processes into the cortex, suggesting a bidirectional requirement for closing the gap between the neuropil and the BBB. In the dorsal ventral nerve cord of third instar larvae, where cortex glial cells are absent, ensheathing glial cells can extend processes into the cortex, contacting and engulfing neuronal cell bodies (Pogodalla et al., 2021). Accordingly, we found that ensheathing glial cells are able to directly contact the blood–brain barrier in the ventral nerve cord, supporting a model in which physical interaction between both glial barriers occurs. This conclusion is based on GFP fragments reconstitution across plasma membranes of both subperineurial- and ensheathing glial cells, however, the extend of contact is affected by the irreversible nature of GFP complementation, and therefore, weak and transient interactions are stabilized (Romei and Boxer, 2019).

Throughout evolution, the emergence of a dedicated barrier to separate the neural tissue from the circulatory system appears to be essential for the acquisition of complex animal behaviors (Abbott, 1992; Dunton et al., 2021). A hallmark of brain architecture lies on the connectivity between the blood–brain barrier, and consequently, the circulatory system with neuronal synapses. In higher vertebrates, this connection is mediated by the versatile action of astrocytes, which extend cell processes to both the BBB and to neurons, including synapses. Our analysis suggests a possible functional parallel to subperineurial glial cells, which could serve as a bridge between the circulatory system and the synaptic regions of the *Drosophila* larval brain. Advancements in labelling techniques and three-dimensional microscopy reconstruction are instrumental in deepening our understanding of neural structures, including the BBB. Therefore, analyzing *Drosophila* glial processes and the potential communication between the BBB and the neural tissue holds the promise of shedding light on the tightly regulated function of the BBB.

## Materials and methods

### *Drosophila* genetics

All *Drosophila* work was conducted according to standard procedures. Fly stocks were kept at room temperature and experimental crosses were maintained at 25°C unless otherwise indicated. We used the following stocks: *mdr65^GMR54C07^-GAL4* (BDSC # 50472) (Jenett et al., 2012; Spéder and Brand, 2014), *moody-GAL4* (Stork et al., 2008), *moody-LexA* (kind gift of S. Schirmeier), *GMR83E12-GAL4* (BDSC #40363) (Jenett et al., 2012; Kremer et al., 2017), *apt^E01^-GAL4* (Contreras and Klämbt, unpublished), *mdr65-LexA* (BDSC #61562), *wor-GAL4* (Albertson et al., 2004), *UAS-mCD8:GFP*, *UAS-mCD8:mCherry*, *UAS-H2B:mRFP* (Langevin et al., 2005), *UAS-CD4:spGFP^1-10^*, *lexAop-CD4:spGFP^11^* (Feinberg et al., 2008), *UAS-Myr-Flag-APEX2-NES* (Rey et al., 2023), *lexAop-mCD8:GFP*, *nrv2:GFP* (BDSC #6828), *moody^ΔC17^* (Bainton et al., 2005; Schwabe et al., 2005), *nrx-IV:GFP^454^* (Edenfeld et al., 2006). UAS-RNAi stocks used: *loco* (VDRC #9248), *mCherry* (BDSC #35785), *moody* (VDRC #1800), *nrx-IV* (VDRC #8353), *Rho1* (VDRC #109420). RNAi experiments were performed at 29°C.

### Immunostaining

Third instar wandering larval brain were dissected in 1x PBS and fixed in 4% formaldehyde in 1x PBS for 20 min. Brains were washed in 0.3% Triton X-100 in 1x PBS for 5 min. 10% normal goat serum was used for blocking. Primary antibodies: rat anti-N-cadherin (CadN, 1:5 catalogue #DN-Ex #8, Developmental Studies Hybridoma Bank), mouse anti-Mdr65 (C219, 1:100, Invitrogen MA1-26528). AlexaFluor conjugated antibodies were used at a concentration of 1:200. Imaging was performed using a Zeiss LSM 880 and LSM 980 confocal microscopes with Fast-Airyscan and 0.4 μm optical sections were acquired every 0.2 μm. z-stacks were reconstructed in three dimension using Imaris 9 (Bitplane). Images, diagrams and figures were assembled using Fiji (Image J, NIH), Adobe Photoshop CC and Adobe Illustrator CC.

### Electron microscopy

Five larval filets were fixed in 4% formaldehyde (FA) in 0.1 M phosphate puffer (P-buffer) for 45 min. Filets were washed 5 times with P-Buffer and incubated with 0.02 M glycine in P-Buffer for 20 min to stop the FA-fixation reaction. Filets were washed 5 times with P-Buffer and incubated in 0.05% diaminobenzidine (DAB) in P-Buffer for 40 min at RT. 0.03% H_2_O_2_ were added for 5–10 min. Filets were washed 3 times with P-Buffer and were fixed with 4%FA and 0.5% glutaraldehyde at RT overnight. FA was replaced by 2% OsO_4_ in P-Buffer for 1 h followed by staining using 2% uranyl acetate (UA) in H_2_O for 30 min in the dark at RT. Subsequently an ethanol gradient (50, 60,70,80, 90, and 96% for 15 min) was performed on ice. Samples were finally dehydrated with 100% ethanol 3 times using a molecular sieve (3Ǻ) and twice propylene oxide for 15 min. After slow EPON infiltration, filets were ultra-flat embedded in Gene Frames (ThermoFisher Scientific) between two layers of ACLAR® film and polymerized at 40°C for 4 days.

For ultrathin sections a 35° ultra knife (Diatome) and an ultramicrotome (Leica EM UC7) were used. Sections of 70 nm thickness were cut and collected on Formvar coated one-slot copper grids. Sections were imaged using an upgraded Zeiss TEM 900 (point electronics) at 80 kV and an iTEM software operated Morada camera (EMSIS, Münster, Germany).

## Data availability statement

The original contributions presented in the study are included in the article/supplementary material, further inquiries can be directed to the corresponding authors.

## Author contributions

EC: Conceptualization, Formal analysis, Investigation, Methodology, Writing – original draft, Writing – review & editing. SK: Investigation, Writing – review & editing. CK: Conceptualization, Funding acquisition, Supervision, Writing – review & editing.
